# Dielectric Properties and Dipole Moment of Edible Oils Subjected to ‘Frying’ Thermal Treatment

**DOI:** 10.3390/foods9070900

**Published:** 2020-07-08

**Authors:** Nataša Šegatin, Tanja Pajk Žontar, Nataša Poklar Ulrih

**Affiliations:** Biotechnical Faculty, University of Ljubljana, 1000 Ljubljana, Slovenia; tanja.pajk@bf.uni-lj.si (T.P.Ž.); natasa.poklar@bf.uni-lj.si (N.P.U.)

**Keywords:** edible oils, dielectric constant, electrical conductivity, dipole moment, polarization, refraction

## Abstract

The dielectric properties of six refined edible oils with different fatty-acid compositions were determined for oils incubated at 180 °C up to 40 h. The oil degradation was evaluated by the dielectric dispersion and dielectric loss in the frequency range from 40 Hz to 2 MHz at 25 °C, refractive index, density, saponification number, and specific absorption coefficient at 232 and 268 nm. The dependence of the dielectric properties on frequency has been evaluated with Corach, Cole–Cole, and the universal power law models, giving the novel strategies for the interpretation of the dielectric spectra of thermally treated oils. The derived parameters—the dielectric constant, the electrical conductivity, the relaxation time *τ* and the exponents α, *p*, and *n*—are discussed with respect to the increased oxidation evidenced by specific absorption coefficients and polar products, as measured by the dielectric constant of the thermally treated oils. The specific refraction, specific polarization, orientation polarization, and dipole moment were determined using Lorenz–Lorentz, Debye and Onsager relationship. All above parameters obtained increased during the thermal treatment, except specific refraction, the electrical conductivity and the relaxation time. The dielectric constant-macroscopic parameter was compared with microscopic parameter polarization and dipole moment; the linear dependence was found to be $R^{2}=0.971$.

## 1. Introduction

Deep-fat frying of food is a one of the standard food processing techniques, where the edible oils are subjected to elevated temperatures [1,2]. Triacylglycerols, also known as triglycerides, are the main components of these oils, accounting for up to 95% while diacylglycerols account for less than 5%. Unsaponifiables account for up to 2% and are composed of phytosterols, carotenoids, tocopherols and phenolics; these compounds exhibit the antioxidative and nutritional properties of the edible oils [3]. The degradation of the oils at elevated temperatures results from oxidation, hydrolysis, polymerization, cis/trans isomerization, conjugation, pyrolysis, and cyclization [4]. With prolonged heating, many different compounds can be formed, such as peroxides, hydroperoxides, free fatty acids, long-chain fatty acids, and other types of esters, monomers, dimers, trimers, and oligomers, which represent the total polar compounds [5]. Some of these products have been reported to be inappropriate or toxic for human consumption [6].

Fatty acids are susceptible to oxidation; their oxidative stability correlates positively with the degree of saturation of fatty acids, the presence of antioxidants metal ions or light [7]. Lipid oxidation is responsible for the greatest reduction in oil quality through the formation of oxidation degradation products. Various aldehydes with 6 to 10 carbon atoms are the most important lipid oxidation products of edible oils which strongly influence the chemical, nutritional, and sensory properties [2,8].

The analyses of abovementioned compounds are carried out with chromatographic methods, but are often time consuming and require environmentally harmful organic solvents [6]. Furthermore, these methods are particularly inappropriate for in situ monitoring of frying oil quality in places like restaurants. To determine the quality of edible oils exposed to elevated temperatures, a various parameters can be evaluated, chemical (e.g., p-anisidine, saponification, peroxide, iodide, free fatty acids) or physical (e.g., density, viscosity, color, refractive index, absorption in the UV, visible or infrared electromagnetic spectra) [9,10,11,12]. Nowadays, the most widely accepted method for determining the degree of degradation of edible oils used in frying is through the total polar compounds, with the most rapid evaluated tests based on changes in the dielectric constant [13,14]. The determination of the dielectric constant of frying oils has also been the subject of detailed studies in terms of new sensors [15,16] or frequency ranges [17,18] and frying procedures [19].

The aim of this study was to determine the changes in dielectric properties of edible oils with different fatty-acid compositions under simulated deep-frying conditions at 180 °C. The thermal degradation of the edible oils was monitored by specific absorption coefficients at 232 and 268 nm, as the detection of the lipid oxidation products—conjugated dienes and trienes and dielectric constants were used to evaluate the total polar compounds. The dielectric spectrum, the dependence of the dielectric dispersion and the loss on frequency were determined in the frequency range from 40 Hz to 2 MHz. Emphasis was given to the parameters derived from the dielectric spectrum according to Corach, Cole–Cole, and the universal power law models: dielectric constant, electrical conductivity, relaxation time and the corresponding exponents. The specific refraction, the specific polarization, and the dipole moment were also evaluated.

## 2. Materials and Methods

### 2.1. Material

Six commercially refined, blanched and deodorized edible oils were purchased in a Slovenian supermarket: coconut oil, corn-germ oil, olive oil, rapeseed oil, sunflower oil, and high-oleic-acid sunflower oil (oleic sunflower oil). The edible oil chosen had different fatty-acid compositions: coconut with high content of saturated fatty acids; oleic sunflower and olive are high in oleic acid; sunflower and corn-germ are high in linoleic; and rapeseed has a high content of linolenic acid. The fatty acid content determined for these oils is given in Appendix A, Table A1.

The oil samples (20 g), distributed into open glass flasks, were incubated in an oven (VO 400; Memmert, Germany) under frying conditions of 180 °C for the same intervals and up to 40 h for all samples to achieve oxidation and degradation. After the thermal treatment, the samples were stored in amber bottles at −20 °C under a nitrogen atmosphere until further analyses. The samples of coconut oil were in the liquid phase during the analysis. All of the chemicals used were of analytical grade.

### 2.2. Methods

#### 2.2.1. Specific Absorption Coefficient at 232 and 268 nm

Spectrophotometric determination of oxidation products in treated oils (dienes and trienes) was on the basis of absorbance at 232 and 268 nm, following a slightly modified version of the International Union of Pure and Applied Chemistry (IUPAC) method [20]. The oil samples were dissolved in the volume of 1-propanol required for the absorbance of the solution to gain between 0.2 and 0.8 a.u., with the absorbance read in quartz cuvettes using a spectrophotometer (8453; Hewlett Packard, Germany). The specific absorption coefficients were calculated. The precision was 1–2%.

#### 2.2.2. Refractive Index

After removal of traces of water, the refractive indices of the oil samples were determined at 25.00 ± 0.02 °C with a refractometer (DUR-W2; Schmidt and Haensch, Germany) equipped with a high-precision water bath (7320; Fluke, Hart Scientific, Everett, WA, USA), according IUPAC 2.102 [20]. The refractometer was calibrated with double-distilled water, and its accuracy was checked with cyclohexane, as ±0.00002.

#### 2.2.3. Density

In accordance with IUPAC 2.101 [20], the densities of the oil samples were determined at 25.00 ± 0.02 °C with accuracy of ±0.00002 g·cm^−3^ using a density meter (PAAR 60; Anton Paar, K. G., Graz, Austria) equipped with an oscillating U-tube (DMA 601) and a thermometer (DT 100-20). The system was calibrated with air and water.

#### 2.2.4. Saponification Value

After 60 min of hydrolysis of 1.8 g of each oil sample with 25.0 mL 0.5-M potassium hydroxide under reflux, the samples were cooled to room temperature and excess of alkali was titrated with standardized 0.5-M hydrochloric acid against phenolphthalein. A blank was also prepared in parallel with the samples [20]. The saponification value is given as mg KOH per g of oil sample. The accuracy was <0.7%.

#### 2.2.5. Dielectric Properties

The dielectric properties of the oil samples were determined by the capacitive method using a precision liquid test fixture (16452A; Agilent Technologies, Japan) and a precision inductance, capacitance and resistance (LCR) meter (E4980A; Agilent Technologies, Japan). After a short compensation of the liquid dielectric test fixture, the parallel capacitance, *C*p, and the equivalent resistance, *R*p, of the samples were measured [21,22]. The equipment was interfaced with a microcomputer for data acquisition and analysis. Both quantities were measured at 155 uniformly distributed frequencies in the range from 40 Hz to 2 MHz at 25.0 ± 0.1 °C with a precision of 0.5% for *C*p and ~2% for *R*p. The temperature control was performed using a high-precision water bath (7320; Fluke, Hart Scientific, Everett, WA, USA) and an external thermometer system (5627A/1502A; Fluke, Hart Scientific, Everett, WA, USA). Before each determination, all traces of water were removed from the oil samples at reduced pressure of 100 mbar and 25 °C. The dielectric dispersion (*ε*′) and dielectric loss factor (*ε*″) for the oil samples were calculated according to Equations (1) and (2):

(1) $$\varepsilon^{\prime }=C_{ps}/K_{c}$$

(2) $$\varepsilon^{\prime \prime }={\left(\omega R_{p}K_{c}\right)}^{-1}$$

where *C*_ps_ is the parallel capacitance (F) of the sample corrected for ‘stray’ capacitance, *K_c_* is the mean cell constant, and *R_p_* is the equivalent parallel resistance (Ω). Following Prevc et al. [18], the cell constant and stray capacitance were determined with data for air and cyclohexane (227048; Sigma-Aldrich, St. Louis, MO, USA). The mean cell constant and stray capacitance were computed by averaging the results determined in the optimal frequency range from 10 to 500 kHz.

### 2.3. Mathematical Models for the Interpretation of the Dielectric Spectra

#### 2.3.1. Corach and Coworkers Model

The relative permittivity ε* of a material can be represented as a complex number:

(3) $$\varepsilon^{*}=\varepsilon^{\prime }-j\varepsilon^{\prime \prime }=\varepsilon^{\prime }-j\left({\varepsilon^{\prime \prime }}_{p}+\frac{\sigma_{0}}{\omega \varepsilon_{0}}\right)$$

where *ε*′ is the dielectric dispersion, as the real part of the relative permittivity, *ε*″ is the dielectric loss factor as the imaginary part of the relative permittivity. The dielectric loss is divided in two terms: ${\varepsilon^{\prime \prime }}_{p}$ is the term representing the absorption and dissipation of the electromagnetic energy associated with the dielectric relaxation processes and $\sigma_{0}/\varepsilon_{0}\omega$ is the conductivity term that describes the dissipation of energy associated with ionic and charge transport, *ω* is the angular frequency (2π*f*), and *j* is an imaginary unit [23].

In the frequency range, where no relaxation processes is seen, the imaginary part associated with the dielectric relaxation processes ${\varepsilon^{\prime \prime }}_{p}$ can be neglected. In this frequency range, the measured *ε*′ and *ε*″ were fitted to Equation (5), as proposed by Corach and coworkers [24] for vegetable oils for frequencies up to 1 MHz:

(4) $$\varepsilon^{*}=\varepsilon_{s}-j\frac{\sigma_{0}}{\varepsilon_{0}\omega }$$

where $\varepsilon_{s}$ is the dielectric dispersion characteristic for the lower frequency range, and is independent of frequency (i.e., the dielectric constant), $\sigma_{0}$ is the electrical conductivity, and $\varepsilon_{0}$ is the permittivity of a vacuum (8.854 × 10^−12^ F m^−1^). The complex fitting was performed with software Origin 8.1.

#### 2.3.2. Cole–Cole model

The decrease in dielectric dispersion, *ε*′, and the increase in dielectric loss, *ε*″, in the dielectric spectrum show the dielectric relaxation processes that can be studied with the Cole–Cole model given in Equation (5) [23]. The Equation (5) can be converted in the function of arcs in Equation (6) [25,26]:

(5) $$\varepsilon^{*}=\varepsilon_{\infty }+\frac{\varepsilon_{s}-\varepsilon_{\infty }}{1+{\left(j\omega \tau \right)}^{\left(1-\alpha \right)}}$$

(6) $${\left(\varepsilon^{\prime }-\frac{\left(\varepsilon_{s}+\varepsilon_{\infty }\right)}{2}\right)}^{2}+{\left(\varepsilon^{\prime \prime }-\frac{\left(\varepsilon_{s}-\varepsilon_{\infty }\right)}{2}\tan\left(\frac{\alpha \pi }{2}\right)\right)}^{2}={\left(\frac{\left(\varepsilon_{s}-\varepsilon_{\infty }\right)}{2}\mathrm{cosec}\left(\frac{\alpha \pi }{2}\right)\right)}^{2}$$

In the Equations, $\varepsilon_{\infty }$ is the dielectric constant characteristic for high frequencies, *τ* is the Cole–Cole mean value of the distribution of relaxation times in the complex material, and α is a constant for a given material, with the values 0 < α < 1. The parameter α was determined with nonlinear implicit curve fitting in OrginPro 2019b, according to Equation (6). The high-frequency dielectric constant $\varepsilon_{\infty }$ was calculated using the refractive index and the Maxwell relationship ($\varepsilon_{\infty }=n_{D}^{2}$). Knowing parameter α, *τ* was calculated using the logarithm form of Equation (7) [27]:

(7) $$\frac{1}{\left(1-\alpha \right)}{\left[\frac{{\left(\varepsilon_{s}-\varepsilon^{\prime }\right)}^{2}+{\varepsilon^{\prime \prime }}^{2}}{{\left(\varepsilon^{\prime }-\varepsilon_{\infty }\right)}^{2}+{\varepsilon^{\prime \prime }}^{2}}\right]}^{1/2}=\omega \cdot \tau$$

#### 2.3.3. Power Law Models

In the frequency range studied, *R*p measurements in liquid foods are often also considered as alternating current conductivity [28]. The alternating current electrical conductivity, $\sigma^{\prime }$, is related to measured *R*p as $\sigma^{\prime }=\varepsilon_{0}/\left(R_{p}K_{c}\right)$ and to dielectric loss factor according to Equation (8) [23]:

(8) $$\sigma^{\prime }=j\omega \varepsilon_{0}\varepsilon^{\prime \prime }$$

For the alternating current electrical conductivity, $\sigma^{\prime }$ power scaling to frequency was used Raju [29] as in Equation (9):

(9) $$\sigma^{\prime }-\sigma_{0}=a\omega^{s}$$

where $\sigma_{0}$ is the electrical conductivity and *a* and *s* are fitting parameters.

In some references [30,31], *R*p and *C*p measurements can also be evaluated as a frequency dependence of the complex alternating current conductivity $\sigma^{*}$, as an intensive property that is derived from admittance *Y* ($\sigma^{*}=Y/K_{c})$ and given in Equation (10) [23,32]:

(10) $$\sigma^{*}=j\omega \varepsilon_{0}\varepsilon^{*}$$

and has been termed the universal dielectric response due to its appearance in many types of disordered systems [23]. The corresponding form of the power law [30,33] is defined in Equation (11):

(11) $$\sigma^{*}-\sigma_{0}=A\omega^{n}$$

where A is an empirical constant, and *n* is the corresponding power-law exponent; both of these are determined from the logarithmic form of Equation (11).

## 3. Results and Discussion

The data from the analyses performed for these oils as before and after the thermal treatment are given in Table 1.

The increases in the absorbances recorded at 232 and 268 nm, which are quantified as the specific absorption coefficients K232 and K268, were used as indicators for monitoring the degree of lipid oxidation. Oxidation of vegetable oils during heating initially results in the formation of hydroperoxides. This process is accompanied by structural rearrangements of the double bonds mainly in polyunsaturated fatty acids, which generates conjugated dienes that result in an increased absorbance at 232 nm [1]. In the further oxidation, secondary oxidation products are formed where the conjugation can be extended to another double bond, the conjugated trienes are formed, which absorb UV light at a wavelength of 268 nm [1]. K232 increased the least for coconut oil (2-fold) followed by olive, oleic sunflower, rapeseed, corn-germ and the most for sunflower oil (17-fold) (Table 1) in accordance to the inherent stabilities (Table A1). The order of the increased values of K268 during the thermal treatment was: coconut oil < oleic sunflower oil < olive oil < rapeseed oil < corn-germ oil < sunflower oil.

In general, the other quality parameters measured (e.g., refractive index (*n*_D_), density (*d*), saponification value (SV)) increased with increasing time of thermal treatment (Table 1). *n*_D_ increased the least, giving the same order as K232: most for linoleic oils (sunflower oil, corn-germ oil, 0.46%) followed by linolenic oil (rapeseed oil, 0.34%), oleic oils (olive oil, oleic sunflower oil, 0.28%) and saturated oil (coconut oil, 0.15%). The oil densities increased the most for rapeseed oil (3.3%), the least for coconut (1.3%). The SV also increased by 3% for oleic sunflower to by 11% for sunflower and for coconut oil by 19%. The values of the *n*_D_ and SV for coconut oil markedly differed from those for all the other oils, which can be explained by the shorter alkyl groups of the fatty acids. The dependence of *n*_D_, *d* and SV for the degree of lipid oxidation, determined as K232 and K268 were illustrated in Supplementary Materials, Figure S1. The slope of the increase in *n*_D_, *d* and SV against K232 and K268 was dependent on the composition of the edible oils with the highest slope for “saturated” coconut oil, followed by “oleic” olive and oleic sunflower and the lowest for “linoleic” corn-germ and sunflower oil.

**Figure 1:**
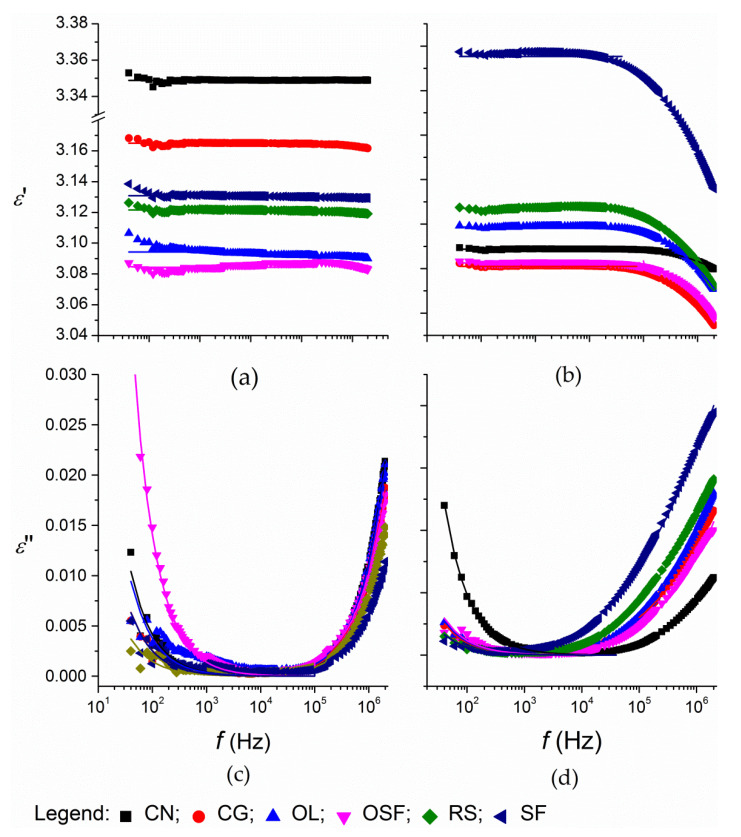
Dependence of the dielectric dispersion, *ε*′ (**a**,**b**) and dielectric loss factor, *ε*″ (**c**,**d**) variations across the lowest (**a**,**c**) and highest (**b**,**d**) dispersion and loss on the frequency range from 40 Hz to 2 MHz, at 25 °C, for the edible oils thermally treated at 180 °C. Lines: fitted values according to Equations (4) and (6). CN, coconut oil; CG, corn-germ oil; OL, olive oil; RS, rapeseed oil; SF, sunflower oil; and OSF, high oleic acid sunflower.

The dielectric dispersion, *ε*′, was independent of frequency up to 1 MHz for the oils before the thermal treatment (Figure 1a), while after thermal treatment, the decrease of *ε*′ began at lower frequencies; for the oils with the highest levels of degradation, this was around 100 kHz (Figure 1b). The dielectric loss, *ε*″, dependence on frequency showed a minimum that was longer lasting for the oils before the thermal treatment compared to after (Figure 1c,d). The shapes of the curves are very similar to those reported for other oils before and after thermal treatments [18,24,34]. The thermal degradation caused increases in *ε*′ at frequencies >1 MHz and pronounced decreases at frequencies approaching 2 MHz. The values of *ε*″ for thermally treated oils were higher at all frequencies, and especially at frequencies >100 kHz.

### 3.1. Corach and Coworkers Model

In the frequency range 40 Hz–100 kHz, *ε*′ decreased by <0.5% and *ε*″ decreased to the minimum for all oil samples investigated. In this frequency range, where no relaxation processes were seen for all oil samples, the measured *ε*′ and *ε*″ were fitted to Equation (4) [24]. The dielectric constant, $\varepsilon_{s}$, and the electrical conductivity, $\sigma_{0}$ subtracted were given in Table 1 and plotted against the K232, *n*_D_ and *d* in Supplementary Materials, Figure S2. The increase in $\varepsilon_{s}$ during the thermal treatment was the highest for sunflower, followed by corn-germ, olive, oleic sunflower oil, and rapeseed, and the lowest for coconut oil. The dependence of $\varepsilon_{s}$ on K232 and K268 (Figure S2a) is the most similar to that of density (Figure S1c). In both cases, a high slope was observed for coconut oil, followed by olive and oleic sunflower, rapeseed and sunflower, and by corn-germ oil. The electrical conductivity $\sigma_{0}$ showed a surprisingly high increase for the saturated and middle chain coconut oil (by 12-fold), compared to the other oils (1–2-fold). Nevertheless, the studied oils can be considered as insulators due to the very low the electrical conductivities in the range of few dozen pS·m^−1^.

Increases in $\varepsilon_{s}$ and $\sigma_{0}$ were a result of increases in polar and ionic species, respectively. Both of these parameters are strongly dependent on the movement of a species within a matrix. For these oils, beside the oxidation processes and the formation of polar compounds, polymerization and increased viscosity of the oils also occurred [10,35]. From the data for $\sigma_{0}$ in Table 1 and Figure S2, it can be seen that the mobility of the ionic species after the prolonged thermal treatment was more hindered in the unsaturated oils, and especially for oleic sunflower oil, compared to the saturated coconut oil, or that fewer ionic species were synthesized in the unsaturated oils. The rotation and alignment with the external electric field of the polar compounds formed during the thermal treatment of all of these vegetable oils were more similar, regardless of the different compositions [13,35]. Consequently, the increase in the dielectric constant remained a better indication of the thermal degradation of these vegetable oils than the electrical conductivity, even if the difficulties of obtaining accurate measurements of electrical conductivity at such low values are ignored. From the Figure S2c, the linear dependence of the electrical constant on density was determined: $\varepsilon_{s}=-19.1+24.3\cdot d$; *R*^2^ = 0.946. This finding might allow determination of degradation of oil with existing instrumentation—density meters.

### 3.2. Cole–Cole Model

The decrease in *ε*′ and *ε*″, according to frequency at frequencies >100 kHz, was more pronounced for samples with a higher degree of degradation (Figure 1). This observation indicates that the relaxation process characteristic for vegetable oils [34] occurred at lower frequencies for the thermally treated oils and/or that the relaxation process was pronounced by the increased content of polar compounds. For the fitting of the *ε*′ and *ε*″ dependence on frequency in the frequency range 100 kHz–2 MHz, the Cole–Cole model (Equations (5) and (6)) was used. In the fitting procedure, the intercepts of the arc in the Cole–Cole plot (Equation (6) with the x axis, (0, $\varepsilon_{\infty }$) and (0, $\varepsilon_{s}$), were added. The fitted values of *ε*′ and *ε*″ obtained by Corach, and Cole–Cole models are given as lines in Figure 1.

**Figure 2:**
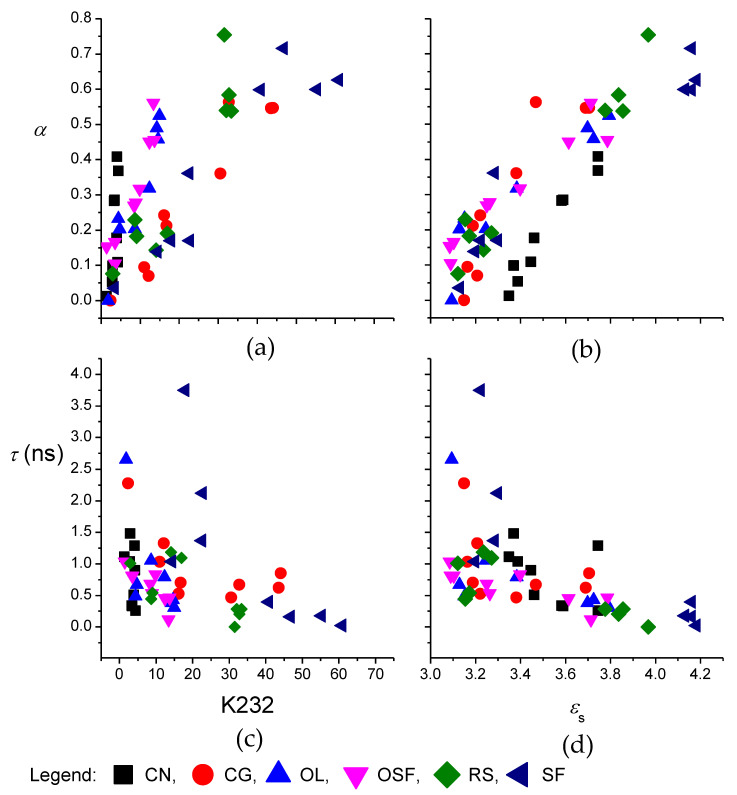
Dependence of the parameters from the Cole–Cole model, as exponent α (**a**,**b**) and mean relaxation time *τ* (**c**,**d**), on the sum of the specific absorption coefficients K232 + K268 (**a**,**c**) and the dielectric constant, *ε_s_* (**b**,**d**) for the edible oils thermally treated at 180 °C. CN, coconut oil; CG, corn-germ oil; OL, olive oil; RS, rapeseed oil; SF, sunflower oil; and OSF, high oleic acid sunflower.

The parameter α and the mean of the distribution of the relaxation times *τ* determined with these procedures were plotted against K232 and *ε_s_* (Figure 2a,b). The values of α increased with increased degradation during the thermal treatment of oils. When α is zero, the dependence of *ε*″ on *ε*′ from the Cole–Cole model (represented by Equation (5)) was reduced to the Debye model, which is used to describe relaxation in a material that shows single relaxation [36]. Such a model appears to be adequate to describe the relaxation process for the oils before the thermal treatment. This shows that the triacylglycerols, which are the main compounds in these refined edible oils, undergo similar relaxation processes in the frequency range studied. During the degradation of the oils at elevates temperatures very diverse compounds were formed, from free fatty acids and oxidation products which increase the content of polar compounds, to polymerization products. Consequently, the compositions of the compounds in the oils following the long thermal treatment became increasingly heterogeneous. The relaxation processes seems to extended over a wider range of frequencies, which is reflected in the distorted semicircle in the Cole–Cole plot as seen from larger values of α [25]. Parameter α increased up to 0.7 for thermal treatment of sunflower oil. The values of *τ* obtained by the procedure described here decreased slightly (nearly independent) with prolonged thermal treatment (Figure 2c,d). The mean *τ* was 8.2 × 10^−10^ s (between 1 × 10^−10^ s and 4 × 10^−9^ s), and the corresponding relaxation frequency was ~200 MHz, which was a lot higher than the measurement frequency range.

**Figure 3:**
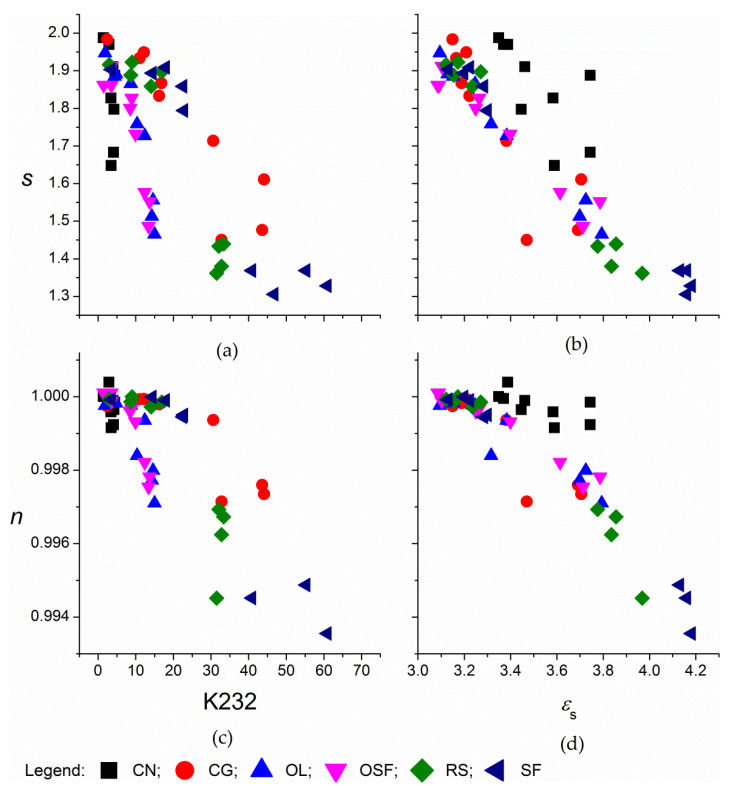
Dependence of exponents *s* from Equation (9) (**a**,**b**) and *n* from Equation (11) (**c**,**d**) on the sum of the specific absorption coefficient K232 (**a**,**c**) and the dielectric constant, *ε_s_* (**b**,**d**) for the edible oils thermally treated at 180 °C. CN, coconut oil; CG, corn-germ oil; OL, olive oil; RS, rapeseed oil; SF, sunflower oil; and OSF, high oleic acid sunflower.

From the relaxation frequencies it is seen that, in the frequency range up to 2 MHz, only a small fraction of the data for the arcs was acquired and for a more precise description of the relaxation process, the data for frequencies >2 MHz was needed. In such a situation, it is possible to use the power law dependence of alternating current electrical conductivity with frequency given in 8. For native oils, the dependence of the logarithm form of Equation (9) showed two regions: at lower frequencies, the slope was low, and for coconut oil even independent of frequency. With the prolonged thermal treatment, these parts were reduced, and for some samples, like the most thermally treated sunflower oil, they practically disappeared. The exponent *s* was determined in the part with the higher slope at frequencies with high correlation coefficients (*R*^2^ > 0.999). The values of *s* for the oils before thermal treatment were between 1.86 and 1.99. With increasing degradation of oils the values *s* decreased (see Figure 3a,b): the smallest decrease was for coconut, to 1.64, the largest for rapeseed and sunflower oil, to 1.38 and 1.31, respectively. The magnitude of *s* is expected to be between 0 and 1. There have also been reports of this being higher, for example for poly(methyl methacrylate), a polymeric species with side ester groups in its structure, at around 1.7 [29]. This strong frequency dispersion was ascribed to hindered rotation of the ester group attached to the main chain [29]. Ester groups are also characteristic for the structure of triacylglycerol molecules.

In terms of the increased oxidation reaction products (K232), the slope was again dependent on the fatty-acid (un)saturation, which was highest for the coconut oil and lowest for the sunflower oil and corn-germ oil (Figure 3a). For the increase in the total polar compounds (*ε_s_*) the slope of the decrease of *s* was very similar across for long-chain oils, while the values for the middle-chain coconut oil were parallel but higher (Figure 3b).

In Figure 3a,b it is also seen that the interpretation of vegetable oil degradation through parameter *n* is possible. The *R*p measurements can even be performed in relatively narrow frequency range for dielectric spectroscopy. The disadvantage of this approach is that only *R*p measurements were considered, while *C*p were ignored. It is necessary to point out again that *R*p measurements for insulating materials like vegetable oils were determined with a greater uncertainty than *C*p.

The power-law dependence for the complex alternating current conductivity σ*, was determined according to the logarithmic form of Equation (11) over the entire frequency range 40 Hz–2 MHz. The value of the exponent *n* for the oils before thermal treatment was 0.9999 ± 0.0001. During the first steps of thermal treatment the exponent decreased only a little, especially for unsaturated corn-germ oil, sunflower oil, and rapeseed oil. For the prolonged thermal treatment, *n* decreased more, to 0.9992 for coconut oil, 0.9975 for oleic sunflower oil, 0.9971 for corn-germ oil and olive oil, and particularly for sunflower oil, to 0.9936 (Figure 3c,d). The dependence of *n* on the formation of polar components, (*ε_s_*) was surprisingly similar across all of the oils with similar alkyl chain length, omitting the double bonds in their structure, although with a different trend for the saturated, middle-chain coconut oil. The disadvantage of this method is that *C*p and *R*p are combined into a complex alternating current conductivity σ*, both measured quantities were not evaluated separately as with *ε*′ and *ε*″.

It has been suggested that for heterogeneous materials, the dielectric response in an electromagnetic field can be described as a network of resistors and capacitors, where exponent *n* can be approximated to the fraction of capacitors [33]. From the magnitude of *n*, it can be concluded that edible oils can be described as the network where capacitors predominate, especially before the thermal treatment.

Interrelationships between the exponents *α*, *s* and *n* are shown in Supplementary Materials, Figure S3.

When a material is placed in an external electric field, relative displacement of the negative and positive charges of the atoms or molecules, i.e., polarization, occurs. For dipolar molecules as triacylglycerols two main contributions occur: permanent orientation and induced polarization. Orientation polarization is characteristic for substances with permanent dipoles that spatially orientate in the external electric field. Induced polarization is composed of two parts: atomic, which arises from the displacement of the atom nucleus, and electric, which includes the elastic displacement of the electron clouds [37,38]. For dipolar materials, electronic polarization is often ascribed as the molar refraction. In the present study, the specific refraction, *r*, was calculated using the Lorenz–Lorentz relationship: [16,39,40]:

(12) $$r=\frac{n_{D}^{2}-1}{n_{D}^{2}+2}\cdot \frac{1}{d}$$

**Figure 4:**
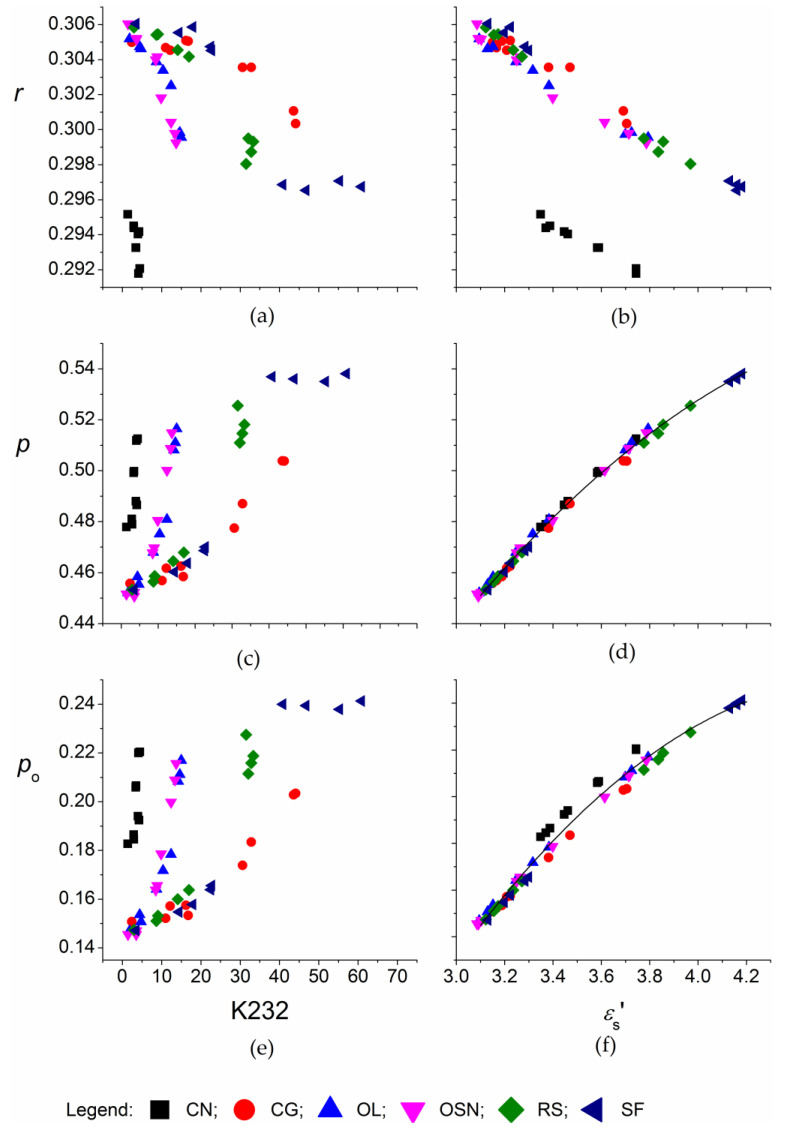
Dependence of the specific refraction, *r* (**a**,**b**), specific polarization, *p* (**c**,**d**), and specific orientation polarization, *p_o_* (**e**,**f**) on the sum of the specific absorption coefficient K232 (**a**,**c**,**e**) and the dielectric constant, *ε_s_* (**b**,**d**,**f**) for the edible oils thermally treated at 180 °C. CN, coconut oil; CG, corn-germ oil; OL, olive oil; RS, rapeseed oil; SF, sunflower oil; and OSF, high oleic acid sunflower.

The specific refraction, *r*, characterizes the electronic polarizability of a unit mass of a substance in the high-frequency electromagnetic field of a light. The values of the *r* of the native edible oils are comparable with the literature data and range from 0.3050 to 0.3061, and for coconut (*r* = 0.2952) (Figure 4a,b) [15,16,39,40]. During thermal treatment, *r* decreased to from 0.2987 to 0.3004, and for coconut to 0.2921, values are similar to some literature reports for vegetable oils [15,39,40]. Regarding the slope of the decrease of K232, it followed the inherent stability, while for $\varepsilon_{s}$, the influence of length of the alkyl chain appears to be predominant.

The total specific polarization, *p*, is related to the static dielectric constant using the Debye relation [15,40]:

(13) $$p=\frac{\varepsilon_{s}-1}{\varepsilon_{s}+2}\cdot \frac{1}{d}$$

We can assume that the specific orientation polarization, *p*_o_, is the difference between the specific polarization and the specific refraction, $p_{o}=p-r$ [16]. For the native oils *p* was 0.453, except for coconut (0.478) and similar to the literature’s data [40,41]. With the increasing degradation, *p* and *p*_o_ increased [15] by 7% for coconut to a 19% for sunflower oil (Figure 4e,f). For the oxidation processes associated with the thermal treatment of the edible oils, the polarizations, *p* and *p*_o_, showed opposite dependence as the specific refraction (Figure 4a,c,e). The dependence of *p* and *p*_o_ on *ε_s_* was very similar for all oils, which compare macroscopic quantities *ε_s_* (and *d*) with microscopic ones ($p=0.084\varepsilon_{s}+0.195,\;R^{2}=0.987$ and $p_{o}=-0.042\varepsilon_{s}{}^{2}+0.39\varepsilon_{s}-0.67,\;R^{2}=0.984)$.

The effective average dipole moment, *µ*, can be calculated using the Onsager relationship, which is appropriate for dipolar and spherical molecules [37]:

(14) $$\frac{\varepsilon_{s}-1}{\varepsilon_{s}+2}\cdot \frac{1}{d}-\frac{n_{D}^{2}-1}{n_{D}^{2}+2}\cdot \frac{1}{d}=p_{o}=\frac{4\pi N_{A}}{9kT\;\bar{M}}\frac{3\varepsilon_{s}\left(n_{D}^{2}+2\right)}{\left(2\varepsilon_{s}+n_{D}^{2}\right)\left(\varepsilon_{s}+2\right)}\mu^{2}$$

where *N_A_* is Avogadro’s number (6.022 × 10^23^ mol^−1^), *k* is the Boltzmann constant (1.3806 × 10^−23^ J·K^−1^), *T* is the absolute temperature, and $\bar{M}$ is the approximate average molecular weight, as determined from the saponification values ($\bar{M}\cdot SV=168300$) [40,42].

**Figure 5:**
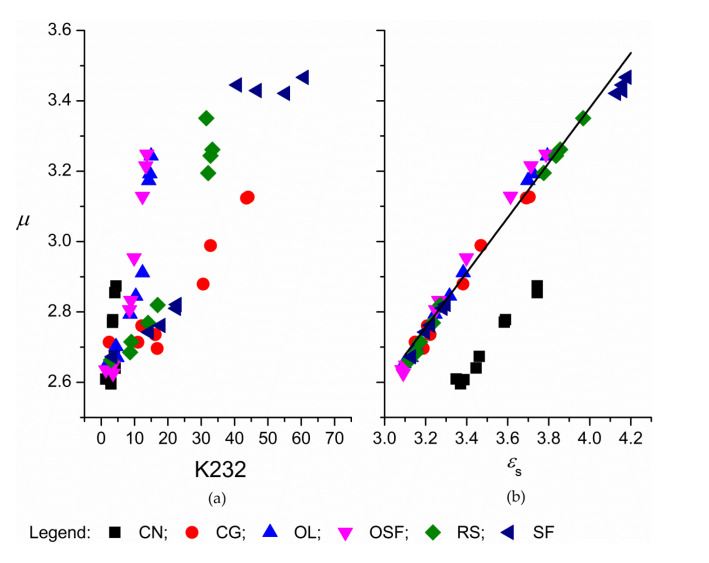
Dependence of the effective dipole moment, *µ*, on the on the sum of the specific absorption coefficients K232 (**a**) and the dielectric constant, *ε_s_* (**b**) for the edible oils thermally treated at 180 °C. CN, coconut oil; CG, corn-germ oil; OL, olive oil; RS, rapeseed oil; SF, sunflower oil; and OSF, high oleic acid sunflower.

The dipole moments determined by the Onsager relation were 5% to 10% higher for the native oils than determined by the Debye relation [40,43], with values from 2.61 to 2.71. For native oils, *µ* were independent of the composition (Figure 5). During thermal treatment, *µ* increased up to 2.874 for coconut and 3.467 for sunflower oil; the increase of *µ* was dependent on the composition, assuming that the increase in *µ* reflected well the degree of degradation of edible oils thermally treated at 180 °C (Figure 5a,b). The dependence on K232 here formed two groups: coconut, oleic sunflower and olive oil and the “linoleic” group: rapeseed, sunflower, corn-germ oil. Regarding the dependence of *µ* on *ε_s_* for coconut oil (middle-length alkyl chains), the higher *ε_s_* were not accompanied by a proportionally higher *µ*, which separated the values for coconut oil. Interrelationships between *µ* and the exponents *α*, *s* and *n* are shown in Figure S3. The average effective dipole moment for the oils with similar lengths of alkyl chains (without coconut) can be ascribed with linear dependences on *ε_s_*, *r, p* and *p*_o_:

$\mu =0.78\varepsilon_{s}+0.26,\;R^{2}=0.987$
$\mu =-88\;r+29.5,\;R^{2}=0.971$
$\mu =9.36p-1.58,\;R^{2}=0.997$
$\mu =8.48{\;p}_{o}+1.41,\;R^{2}=0.997$

## 4. Conclusions

For edible oils with different fatty-acid compositions which have been subjected to a thermal treatment at 180 °C for up to 40 h, the degree of degradation has been determined on the basis of the several physical properties. The most comprehensive investigations were carried out on the interactions of the treated oils with radio waves in the range from 40 Hz to 2 MHz. The dielectric dispersion and dielectric loss were evaluated by the Corach and Cole–Cole models, and the electrical conductivity by the power laws. Finally, the specific refraction, the specific polarization and the average effective dipole moments were determined. All of these parameters derived were discussed in terms of the degradation of the thermally treated oils, which was determined by the specific absorption coefficients reflecting the primary and secondary oxidation products formed and the dielectric constants reflecting the formation of the polar compounds in these edible oils.

The measured and derived physicochemical parameters mostly increased with increasing degree of oxidation in the thermally treated oils, i.e., refractive index, density, saponification value, dielectric constant, Cole–Cole exponent α, specific polarization and, finally, the average effective dipole moment. These included the exception of the specific refraction and power laws’ exponents *n* and *s*, which decreased, and the Cole–Cole mean relaxation time and direct current electrical conductivity, which were independent of the absorption coefficients. The differences resulting from the degradation of the edible oils were up to 33% for the dielectric constant, and up to 70% for the specific orientation polarization and the average effective dipole moment. With the high content of saturated, middle-chain fatty acids in the structure of saturated coconut oil, the trends of the different physical properties on the degree of oxidation generally differed from the other edible oils with higher fatty-acid lengths. This was the case for refractive indices, direct current electrical conductivity, and specific refraction. For the dielectric constant, density, Cole–Cole and universals law exponents α, *n* and *s*, the edible oils were grouped according to the saturation/unsaturation of their fatty acids: saturated coconut, oleic olive and sunflower, polyunsaturated corn-germ, rapeseed and sunflower oils.

Based on the dependence of the total polar contents, the following group-based particularly for these edible oils were defined: (i) all of these edible oils showed very similar dependence on specific and orientation polarization; (ii) the saturated coconut oil differed from all of the other edible oils in terms of direct current electrical conductivity, refractive index, specific refraction, universals law exponents *n* and *s*, and average effective dipole moment; (iii) the edible oils differed the most according to fatty-acids composition and inherent stability for Cole–Cole exponent *α* and (iv) the Cole–Cole mean relaxation times were independent of the type of edible oil.

The Cole–Cole and the universal power law models were used for the first time for the interpretation of the dielectric spectra in range of radiofrequencies for thermally treated edible oils at simulated frying temperature. Furthermore, the macroscopic parameter–dielectric constant was compared with microscopic parameters specific refraction, polarization and dipole moment and linear dependence was found for all thermally treated edible oils (*R*^2^ > 0.971).

## Figures and Tables

**Table 1:** Parameters measured for the edible oils before and during the thermal treatment, determined at 25 °C.

Edible Oil	K232	K268	Saponification Value (mg/g)	Refractive Index *n*_D_	Density *d/*(g/cm^3^)	Dielectric Constant ^a^ *ε*_s_	Conductivity σ_0_/(nS/m)
Coconut	1.39	0.12	252.2	1.45480	0.91884	3.3488 ± 0.0001	0.023 ± 0.001
	2.87	0.31	258.9	1.45500	0.92126	3.3873 ± 0.0001	0.046 ± 0.001
	2.94	0.32	258.0	1.45496	0.92155	3.3702 ± 0.0002	0.037 ± 0.004
	3.97	0.46	258.3	1.45540	0.92343	3.4614 ± 0.0001	0.080 ± 0.001
	4.21	0.44	262.1	1.45544	0.92306	3.4462 ± 0.0001	0.081 ± 0.001
	3.40	0.62	258.8	1.45596	0.92685	3.5834 ± 0.0001	0.180 ± 0.001
	3.47	0.63	258.4	1.45598	0.92688	3.5895 ± 0.0001	0.268 ± 0.001
	4.10	1.11	265.3	1.45694	0.93326	3.7437 ± 0.0002	0.270 ± 0.003
	4.43	1.25	262.2	1.45678	0.93210	3.7440 ± 0.0002	0.291 ± 0.002
Corn-germ	2.4	1.55	186.8	1.47054	0.91569	3.1491 ± 0.0001	0.024 ± 0.001
	11.0	3.83	189.0	1.47107	0.91749	3.1653 ± 0.0003	0.021 ± 0.003
	12.2	4.23	189.5	1.47121	0.91818	3.2082 ± 0.0001	0.030 ± 0.002
	16.2	6.09	193.5	1.47323	0.91986	3.2222 ± 0.0001	0.023 ± 0.001
	16.8	5.72	193.3	1.47322	0.91996	3.1882 ± 0.0001	0.031 ± 0.001
	30.6	7.32	196.3	1.47464	0.92688	3.3819 ± 0.0001	0.034 ± 0.002
	32.8	7.77	194.2	1.47473	0.92702	3.4695 ± 0.0003	0.032 ± 0.004
	43.6	9.87	201.3	1.47694	0.93841	3.6909 ± 0.0005	0.058 ± 0.008
	44.1	9.97	201.9	1.47723	0.94114	3.7050 ± 0.0006	0.056 ± 0.010
Olive	1.8	0.15	189.6	1.46709	0.90935	3.0944 ± 0.0002	0.021 ± 0.003
	4.4	0.93	192.4	1.46740	0.91121	3.1518 ± 0.0001	0.035 ± 0.001
	4.7	0.98	192.7	1.46743	0.91160	3.1290 ± 0.0001	0.033 ± 0.001
	8.7	1.12	194.2	1.46808	0.91493	3.2462 ± 0.0001	0.037 ± 0.001
	10.4	1.22	197.6	1.46840	0.91694	3.3161 ± 0.0001	0.044 ± 0.001
	12.4	1.40	197.4	1.46905	0.92069	3.3834 ± 0.0001	0.036 ± 0.002
	14.6	2.04	202.0	1.47054	0.93144	3.7249 ± 0.0004	0.049 ± 0.007
	14.2	2.46	201.1	1.47066	0.93196	3.6984 ± 0.0007	0.050 ± 0.009
	15.0	2.49	202.5	1.47126	0.93354	3.7932 ± 0.0007	0.061 ± 0.010
Rapeseed	2.9	0.80	189.2	1.47096	0.91388	3.1215 ± 0.0001	0.014 ± 0.001
	8.7	2.27	191.3	1.47134	0.91575	3.1546 ± 0.0001	0.008 ± 0.001
	9.0	2.38	190.2	1.47145	0.91581	3.1729 ± 0.0001	0.010 ± 0.001
	14.1	3.36	192.2	1.47180	0.91913	3.2354 ± 0.0001	0.012 ± 0.001
	16.9	3.44	190.6	1.47212	0.92082	3.2715 ± 0.0001	0.011 ± 0.001
	31.5	6.23	202.6	1.47597	0.94629	3.9681 ± 0.0012	0.055 ± 0.017
	33.3	6.33	203.1	1.47536	0.94120	3.8557 ± 0.0001	0.052 ± 0.012
	32.1	5.59	202.8	1.47526	0.94045	3.7755 ± 0.0001	0.054 ± 0.012
	32.8	6.15	202.0	1.47602	0.94419	3.8355 ± 0.0011	0.050 ± 0.016
Sunflower	3.5	2.06	187.7	1.47275	0.91618	3.1309 ± 0.0001	0.014 ± 0.001
	14.4	3.51	188.7	1.47335	0.91870	3.1983 ± 0.0001	0.005 ± 0.001
	17.9	3.87	190.3	1.47349	0.91798	3.2238 ± 0.0001	0.003 ± 0.001
	22.8	4.11	192.9	1.47410	0.92305	3.2996 ± 0.0003	0.044 ± 0.005
	22.5	4.08	192.1	1.47414	0.92244	3.2847 ± 0.0001	0.015 ± 0.002
	40.8	9.12	206.4	1.47918	0.95553	4.1602 ± 0.0017	0.035 ± 0.014
	55.3	9.12	206.7	1.47892	0.95439	4.1301 ± 0.0016	0.034 ± 0.013
	46.8	10.35	207.8	1.47948	0.95709	4.1604 ± 0.0015	0.047 ± 0.025
	55.3	11.98	205.4	1.47948	0.95641	4.1810 ± 0.0014	0.036 ± 0.018
Sunflower:	1.4	0.49	190.1	1.46776	0.90790	3.0849 ± 0.0001	0.078 ± 0.002
high oleic	3.5	0.87	191.6	1.46816	0.91107	3.0905 ± 0.0001	0.075 ± 0.001
	3.6	0.82	190.6	1.46815	0.91115	3.1028 ± 0.0001	0.075 ± 0.002
	8.5	0.85	192.2	1.46898	0.91607	3.2494 ± 0.0001	0.075 ± 0.001
	8.9	0.91	191.0	1.46894	0.91550	3.2638 ± 0.0001	0.077 ± 0.001
	9.9	0.93	192.4	1.47014	0.92465	3.3986 ± 0.0002	0.053 ± 0.002
	13.7	1.82	200.5	1.47164	0.93513	3.7861 ± 0.0005	0.049 ± 0.007
	12.4	1.25	196.4	1.47119	0.93070	3.6136 ± 0.0004	0.043 ± 0.006
	13.4	1.41	196.7	1.47167	0.93348	3.7132 ± 0.0005	0.042 ± 0.008

^a^ For fitting procedure according to Corach model (Equation (4)) $R^{2}>0.9999$.

## Supplementary Materials

The following are available online at https://www.mdpi.com/2304-8158/9/7/900/s1, Figure S1: Dependence of the refractive index, density, and saponification value on the specific absorption coefficients K232 and K268 for the edible oils thermally treated at 180 °C; Figure S2: Dependence of the dielectric constant, and electrical conductivity, on the specific absorption coefficients K232, refractive index and density for the edible oils thermally treated at 180 °C; Figure S3: Dependence of the effective dipole moment on the exponents α, *n* and *s* and interdependence of exponents: *s* on α and *n* and exponent *n* on α.

## Appendix A

### Fatty Acid Composition—Analytical Procedure

The fatty acid composition in Table A1 was determined on GC as fatty acid methyl esters (FAMEs) in accordance with IUPAC 2.301 and 2.302. The FAMEs were quantified on an Agilent 6890N gas chromatograph, with a flame ionization detector and an Agilent 7683 autosampler (Agilent Technologies; Palo Alto, CA, USA) on an SPB PUFA; a 30 m × 0.25 mm × 0.2 μm column (Supelco) was used. Heptadecanoic acid (Sigma H 3500) was used as an internal standard; the fatty acids were identified by methyl ester standards mixture obtained from Supelco, (Bellefonte, Pennsylvania, USA). [20].

**Table A1:** Fatty acid composition of edible oils (%) and their inherent stabilities.

Edible Oil	Fatty Acid Composition (%)							
	C10:0	C12:0	C14:0	C16:0	C18:0	C18:1	C18:2	C18:3
Coconut	7.89 ± 0.85	40.0 ± 0.92	15.99 ± 0.34	8.25 ± 0.14	2.34 ± 0.04	5.94 ± 0.10	1.31 ± 0.02	0.040 ± 0.005
Corn germ	nd	nd	0.12 ± 0.02	9.93 ± 0.05	1.43 ±0.01	27.22 ± 0.15	48.21 ± 0.26	0.97 ± 0.04
Olive	nd	nd	0.009 ± 0.001	11.33 ± 0.01	2.56 ± 0.01	69.81 ± 0.02	6.74 ± 0.01	0.68 ± 0.02
Rapeseed	nd	nd	0.048 ± 0.006	4.46 ± 0.07	1.26 ± 0.04	53.93 ± 0.25	21.05 ± 0.06	6.45 ± 0.01
Sunflower	nd	nd	0.073 ± 0.001	5.56 ± 0.02	2.60 ± 0.01	25.87 ± 0.13	53.09 ± 0.25	0.301 ± 0.001
Sunflower: high oleic	nd	nd	0.152 ± 0.002	3.90 ± 0.02	2.09 ± 0.03	75.81 ± 0.33	8.10 ± 0.03	0.205 ± 0.003

nd, not detected.
