# Supplementary material for: Dielectric Properties and Dipole Moment of Edible Oils Subjected to ‘Frying’ Thermal Treatment
Source: Foods. 2020 Jul 8;9(7):900. doi: 10.3390/foods9070900 (PMC7404983; doi:10.3390/foods9070900)

**Supplementary Materials - Caption**

**Figure S1.** Dependence of the refractive index, $n_D$ (**a, b**), density, $d$ (**c, d**), and saponification value, SV (**e, f**) on the specific absorption coefficients K232 (**a, c, e**) and K268 (**b, d, f**) for the edible oils thermally treated at 180 °C. CN, coconut oil; CG, corn-germ oil; OL, olive oil; RS, rapeseed oil; SF, sunflower oil; and OSF, high oleic acid sunflower.

**Figure S2.** Dependence of the dielectric constant, $\varepsilon_s$ (**a, b, c**) and electrical conductivity, $\sigma_0$ (**d, e, f)** on the specific absorption coefficients K232 (**a, d**), refractive index $n_D$ (**b, e**) and density $d$ (**c, f**) for the edible oils thermally treated at 180 °C. CN, coconut oil; CG, corn-germ oil; OL, olive oil; RS, rapeseed oil; SF, sunflower oil; and OSF, high oleic acid sunflower, line regresion.

**Figure S3.** Dependence of the effective dipole moment, $\mu$, on the exponents $\alpha$, $n$ and $s$ (**a, b, c**), and interdependence of exponents: $s$ on $\alpha$ and $n$ (**d, e**) and exponent $n$ on $\alpha$ (**f**), lines – regression.

## Supplementary Materials

**Figure S1.** Dependence of the refractive index, $n_D$ (**a, b**), density, *d* (**c, d**), and saponification value, SV (**e, f**) on the specific absorption coefficients K232 (**a, c, e**) and K268 (**b, d, f**) for the edible oils thermally treated at 180 °C. CN, coconut oil; CG, corn-germ oil; OL, olive oil; RS, rapeseed oil; SF, sunflower oil; and OSF, high oleic acid sunflower.

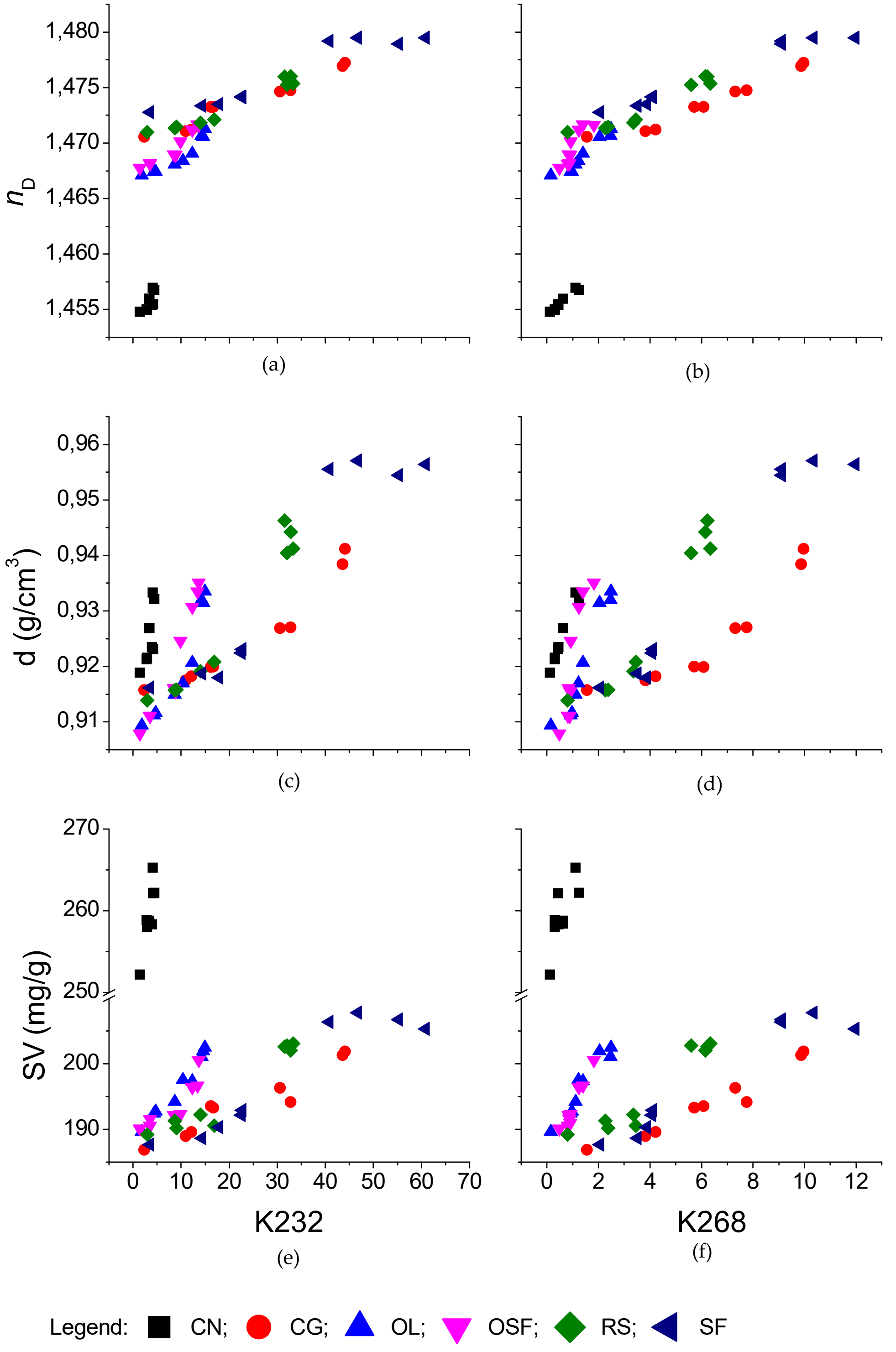

**Figure S2.** Dependence of the dielectric constant, $\varepsilon_s$ (**a, b, c**) and electrical conductivity, $\sigma_0$ (**d, e, f**) on the specific absorption coefficients K232 (**a, d**), refractive index $n_D$ (**b, e**) and density $d$ (**c, f**) for the edible oils thermally treated at 180 °C. CN, coconut oil; CG, corn-germ oil; OL, olive oil; RS, rapeseed oil; SF, sunflower oil; and OSF, high oleic acid sunflower - line regresion.

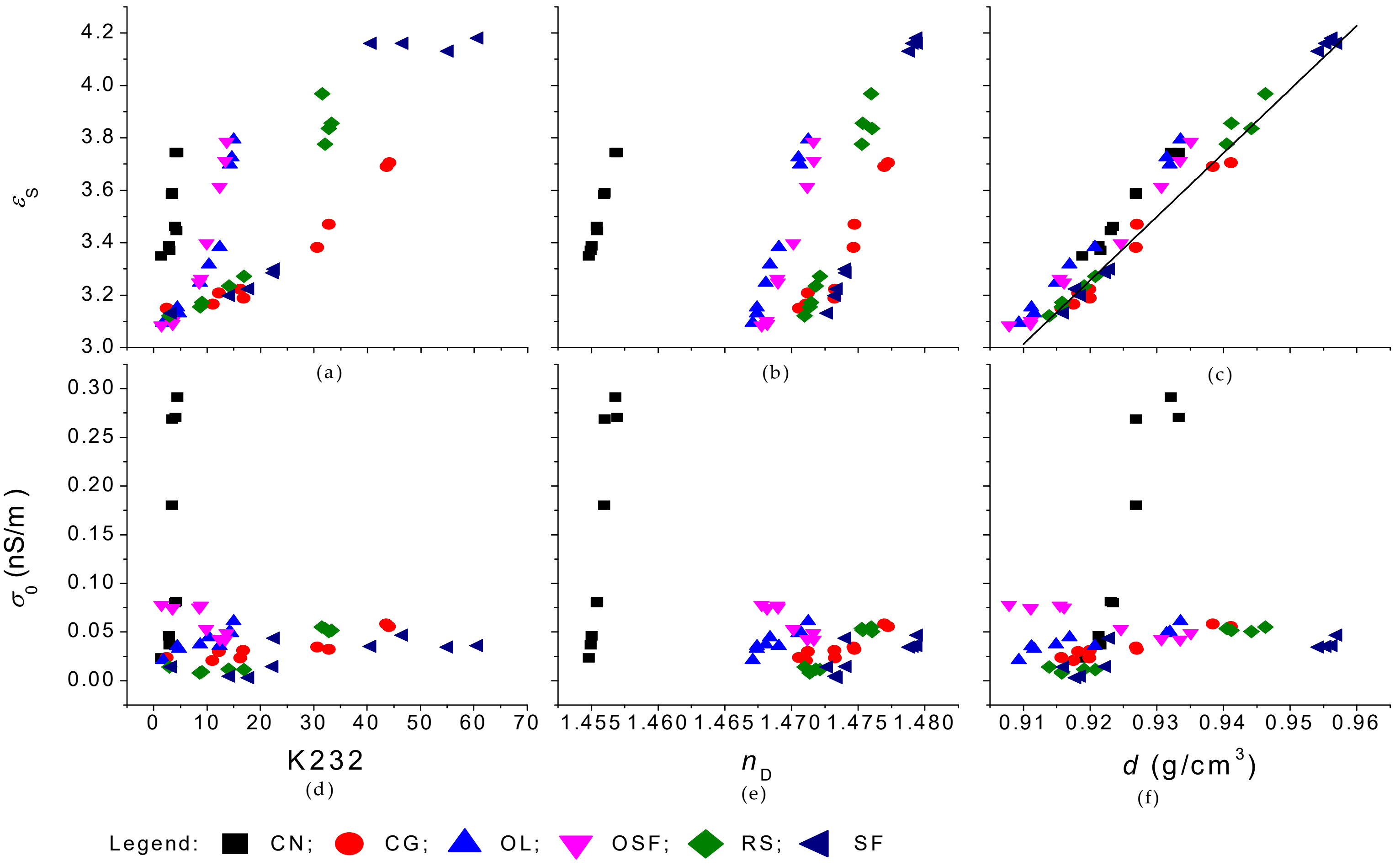

**Figure S3.** Dependence of the effective dipole moment, $\mu$, on the exponents $\alpha$, $n$ and $s$ (**a, b, c**), and interdependence of exponents: $s$ on $\alpha$ and $n$ (**d, e**) and exponent n on $\alpha$ (**f**), lines –regression.

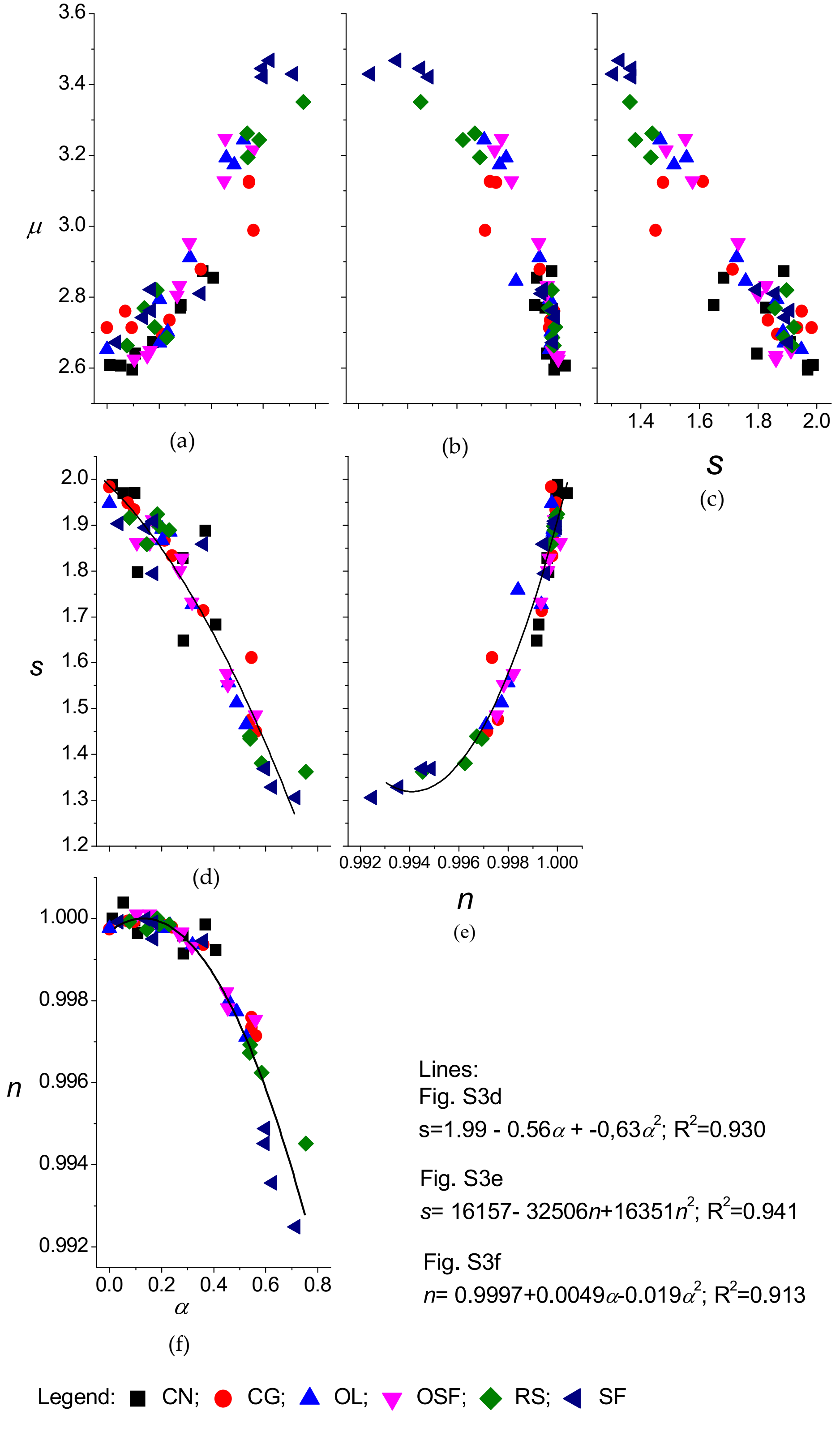

Supplement: Supplementary file 1 [file foods-09-00900-s001.pdf]
